# Rapid Endocrine Remission After ZAP-X Gyroscopic Radiosurgery for Cushing’s Disease: A Case Report

**DOI:** 10.7759/cureus.96636

**Published:** 2025-11-11

**Authors:** Alper Kahvecioglu, Selin Tekin, Seda Hanife Oguz, Mustafa Berker, Selcuk Dagdelen, Gozde Yazici

**Affiliations:** 1 Radiation Oncology, Hacettepe University Faculty of Medicine, Ankara, TUR; 2 Endocrinology, Hacettepe University Faculty of Medicine, Ankara, TUR; 3 Neurosurgery, Hacettepe University Faculty of Medicine, Ankara, TUR

**Keywords:** cushing’s disease, pituitary adenoma management, radiotherapy (rt), stereotactic ablative radiation, stereotactic radio surgery (srs), stereotactic radiosurgery srs, zap-x radiosurgical system

## Abstract

Cushing’s disease is a rare but potentially life-threatening disorder caused by excessive adrenocorticotropic hormone (ACTH) secretion from a pituitary adenoma. Although transsphenoidal surgery remains the first-line treatment, radiotherapy (RT) can provide effective local and hormonal control in patients with persistent or recurrent disease; however, endocrine remission typically occurs only after several months or even years. To our knowledge, we report the first documented case of an exceptionally rapid hormonal remission following gyroscopic stereotactic radiosurgery (SRS) using the self-shielding ZAP-X system (ZAP Surgical Inc., San Carlos, CA, USA) in a patient with recurrent Cushing’s disease. The patient received a single-fraction dose of 25 Gy prescribed to the 57% isodose line. Remarkably, ACTH and cortisol levels normalized within one month after SRS, accompanied by a striking improvement in clinical symptoms and no treatment-related toxicity. This case highlights the potential of the ZAP-X gyroscopic radiosurgery platform to achieve rapid biochemical control in ACTH-secreting pituitary adenomas and suggests that the unique dose distribution characteristics of this novel technology may contribute to accelerated endocrine responses.

## Introduction

Cushing's disease stems from an excess production of adrenocorticotropic hormone (ACTH) by a pituitary adenoma, leading to elevated cortisol levels and symptoms resembling Cushing's syndrome [1]. Untreated or inadequately managed hypercortisolism is associated with substantial morbidity and elevated mortality rates for patients with Cushing’s syndrome. While transsphenoidal surgery is frequently considered the preferred initial treatment approach, radiotherapy (RT) can also be contemplated, either as a standalone option for patients ineligible for surgery or as part of a multidisciplinary approach in instances where an adequate response is not attained following surgery. Conventional fractionated RT (CFRT), fractionated stereotactic radiosurgery (F-SRS), and stereotactic radiosurgery (SRS) have all been employed in the treatment of Cushing's disease, yielding comparable disease control rates ranging from 46% to 100% [2-4]. However, unlike surgery, the response to RT may require time to manifest, and in certain instances, this duration can extend over months or years [4]. Sheehan et al. [5] indicated that the cure rate after RT in patients with Cushing's disease was 34% in the first year, increasing to 78% by the fifth year. Although CyberKnife (Accuray Inc., Sunnyvale, CA, USA), Gamma Knife (Elekta AB, Stockholm, Sweden), and Linear Accelerator (LINAC)-based systems are all available for F-SRS and SRS, technological advancements are increasing the options. We have integrated the relatively new vault-free, frameless, gyroscopic radiosurgery system, ZAP-X (ZAP Surgical Inc., San Carlos, CA, USA), into our department and have begun using it for cranial F-SRS and SRS treatments [6,7]. To our knowledge, this report introduces the initial case of a patient diagnosed with Cushing's disease who underwent gyroscopic SRS with the ZAP-X system, experiencing an unexpectedly rapid endocrine response following SRS.

## Case presentation

A 48-year-old female with an unremarkable medical history except for hyperlipidemia underwent investigation in 2018 due to symptoms and findings consistent with Cushing's syndrome, leading to the detection of hypercortisolism. Her 24-hour urinary free cortisol and ACTH levels were 75 µg/day and 32 pg/mL, respectively. Serum cortisol remained unsuppressed following the 1 mg dexamethasone suppression test (DST), measuring 15.7 mcg/dL. On the magnetic resonance imaging (MRI) of the pituitary gland, a 4.5x3 mm microadenoma was detected in the left half of the adenohypophysis. Following the referral to the neurosurgery department, the patient underwent tumor resection via transsphenoidal endoscopic surgery in December 2018. The pathology resulted in a corticotroph pituitary adenoma. All her symptoms and signs related to Cushing’s disease resolved after surgery, and postoperative MRI showed no residual tumor. The patient was placed under observation without additional treatment. During follow-up visits, the patient remained asymptomatic for approximately five years.

**Figure 1:**
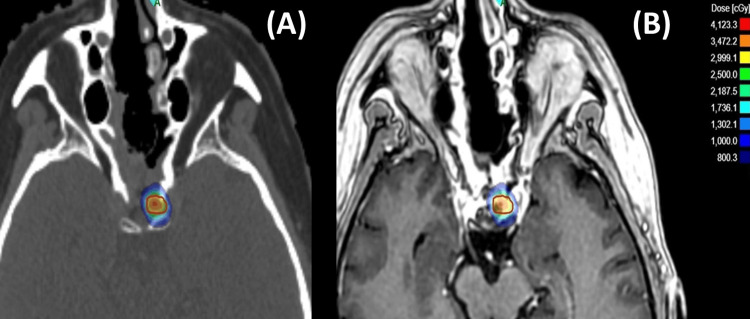
Dose distribution of the gyroscopic stereotactic radiosurgery plan for pituitary adenoma The image shows the three-dimensional dose distribution generated using the ZAP-X system (ZAP Surgical Inc., San Carlos, CA, USA). The prescription dose of 25 Gy to the 57% isodose line is illustrated. A: planning computed tomography (CT) scan showing the isodose distribution around the target; B: planning magnetic resonance imaging (MRI) fused with CT for target delineation.

**Figure 2:**
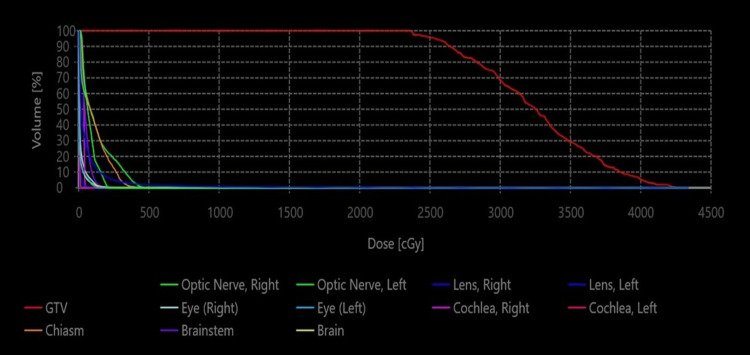
Dose-volume histogram (DVH) of the gyroscopic stereotactic radiosurgery plan The DVH demonstrates a steep dose fall-off beyond the target margins, with minimal exposure to the optic nerves, optic chiasm, and brainstem, confirming optimal dose conformity and effective sparing of organs at risk. Within the gross tumor volume (GTV), the dose distribution is intentionally inhomogeneous, with a hot spot centrally located to ensure adequate tumor coverage and biological effectiveness.

In September 2023, the patient presented with complaints of proximal muscle weakness, irregular menstruation, and Cushingoid appearance. Upon evaluation, hypercortisolism was detected once again. On the pituitary MRI, no residual or recurrent lesion was observed. Subsequently, the patient underwent a second transsphenoidal surgery, but the pathology result did not reveal tissue consistent with a pituitary adenoma. In January 2024, upon initial presentation to our center, the patient's ACTH level was 29.8 pg/mL, 24-hour urinary free cortisol was 442 µg/day, and serum cortisol following a 1 mg DST was 19 mcg/dL. The levels of the remaining anterior pituitary hormones were within normal ranges. The patient, who continued to exhibit symptoms consistent with Cushing's syndrome, underwent another pituitary MRI. At this point, it was discovered that there was a recurrent lesion measuring 2x1 mm on the left half of the adenohypophysis. Pasireotide (0.6 mg once daily) was initiated for persistent hypercortisolism but was discontinued due to frequent diarrhea and a widespread allergic skin reaction. The patient was then evaluated by the multidisciplinary neuro-oncology tumor board at our hospital, which recommended RT as the next step. SRS was selected as the RT technique due to the tumor’s small size and its lack of proximity to critical structures such as the optic chiasm. A simulation computed tomography (CT) scan with a 1 mm axial slice thickness was conducted with the patient in the supine position. Intravenous contrast and a thermoplastic mask were utilized to ensure better visualization and precise immobilization. Gross tumor volume (GTV) was delineated as the macroscopic tumor volume according to the MRI, which was performed a few days before SRS. A planning target volume (PTV) was not generated for this case. The prescription dose was 2500 cGy in a single fraction to the 57% isodose (Figure 1). Dose-volume histogram (DVH) was presented in Figure 2. The SRS plan was generated with the integrated ZAP-X treatment planning software (version 1.8.58.12369), and detailed parameters of the plan were presented in Table 1.

**Table 1:** Detailed parameters of the gyroscopic radiosurgery plan Dmax: maximum dose; Dmean: mean dose; Dmin: minimum dose; GTV: gross tumor volume; mm: millimeter; min: minute

Parameters	Values
Volume (GTV)	0.13 cm^3^
Prescription dose & isodose	2500 cGy & 57.6%
Coverage	95.68%
Homogeneity index	1.74
New conformity index	1.48
Gradient index	3.58
GTV Dmean	3249 cGy
GTV Dmax	4340 cGy
GTV Dmin	2364 cGy
Optic chiasm (Dmax)	452 cGy
Left optic nerve (Dmax)	480 cGy
Right optic nerve (Dmax)	212 cGy
Brainstem (Dmax)	233 cGy
Number of beams	128
Number of isocenters	3
Monitor units	16.121
Collimator thicknesses	4 & 4 & 5 mm
Treatment delivery time	33 min

**Figure 3:**
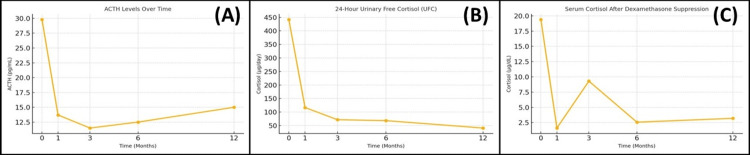
Temporal changes in hormonal parameters following gyroscopic stereotactic radiosurgery A: adrenocorticotropic hormone (ACTH) levels showed a rapid decline within the first month after treatment, remaining suppressed throughout follow-up; B: twenty-four-hour urinary free cortisol (UFC) demonstrated a similar sharp reduction after radiosurgery, indicating early biochemical response; C: serum cortisol levels after dexamethasone suppression normalized by the first month and remained within the physiological range during subsequent evaluations, consistent with sustained hormonal remission.

**Figure 4:**
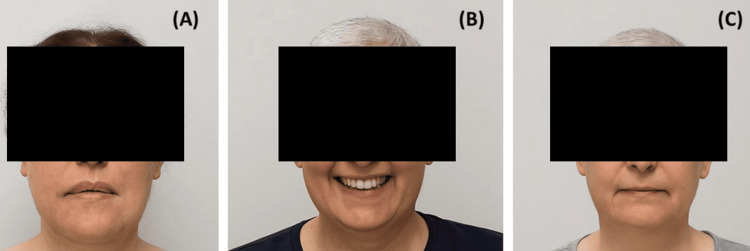
Facial appearance before and after gyroscopic stereotactic radiosurgery (SRS) The images illustrate the patient’s appearance at the time of initial diagnosis (A), before SRS (B), and after the procedure (C).

The treatment was well tolerated, and a marked biochemical response was observed one month after SRS, with ACTH and 24-hour urinary free cortisol levels decreasing to 14.2 pg/mL and 116 µg/day, respectively. Serum cortisol following a 1 mg DST was suppressed to 1.6 µg/dL. Concurrently, there was a noticeable improvement in the clinical signs and symptoms of Cushing’s disease. The patient was subsequently followed with regular clinical assessments at three-month intervals for one year. Throughout the follow-up period, ACTH, 24-hour urinary free cortisol, and post-DST serum cortisol levels remained near-normal (Figure 3). Levels of other anterior pituitary hormones were within normal limits. The patient’s biochemical parameters, including ACTH, urinary free cortisol, and serum cortisol levels before and after SRS, are summarized in Table 2. At the three-month post-SRS MRI, the lesion was found to be radiologically stable. However, the patient reported a subjective improvement in proximal muscle weakness beginning one month after treatment. No SRS-related toxicity was observed during the follow-up period, and partial regression of the Cushingoid phenotype was documented (Figure 4).

**Table 2:** Summary of laboratory findings before and after gyroscopic stereotactic radiosurgery ACTH: adrenocorticotropic hormone; UFC: urinary free cortisol; DST: dexamethasone suppression test; SRS: stereotactic radiosurgery

Parameter	Unit	Reference Range	At Initial Diagnosis (2018)	Recurrence (Jan 2024, before SRS)	1 Month After SRS	3 Months After SRS	6 Months After SRS	12 Months After SRS
ACTH	pg/mL	7.2 – 63.3	32	29.8	14.2	11.0	12.5	15.0
24-hour UFC	µg/day	20 – 90	75	442	116	65	55	45
Serum cortisol after 1 mg DST	µg/dL	< 1.8 (suppressed)	15.7	19.0	1.6	9.0	2.5	3.0

## Discussion

To our knowledge, we report a rapid endocrine response observed in the first patient with Cushing’s disease treated using the ZAP-X gyroscopic radiosurgery system. Despite the patient having a significantly high 24-hour urinary free cortisol level before SRS, there was a considerable decrease within a short period after SRS.

Both surgical and medical treatments, along with RT, are viable approaches for managing pituitary adenomas. Approximately 70% of pituitary adenomas are associated with syndromes characterized by excessive hormone secretion, with the most common types producing prolactin, growth hormone, and ACTH [8]. Unlike non-secreting adenomas, the treatment goal for secreting adenomas extends beyond local tumor control to include the management of endocrinopathies. Although transsphenoidal surgery is commonly regarded as the first-line treatment, RT may also be considered, either as a primary modality in patients who are not surgical candidates or as part of a multimodal strategy when surgical outcomes are suboptimal. CFRT, F-SRS, and SRS have all been employed in treating patients with Cushing's disease, yielding comparable disease control rates [9]. CFRT may be preferred, particularly for larger tumors or those located near organs at risk (OAR). In appropriately selected cases, advanced techniques such as F-SRS and SRS can shorten treatment duration and enable dose escalation within the tumor while providing a rapid dose fall-off outside the target volume. While RT can effectively control local tumor growth, its success in addressing endocrinopathies is typically more limited. In a systematic review, the rates of local tumor control and endocrine control for Cushing's disease were reported as 92% and 48%, respectively [9]. Additionally, the radiation doses required for tumor control and endocrine response vary from each other [10]. While SRS doses ranging from 12 to 20 Gy typically achieve adequate local tumor control, especially in non-secreting adenomas, it has been observed that endocrine response rates improve at marginal doses around 30 Gy [11,12]. However, administering high doses can be challenging due to the presence of OAR, such as the optic apparatus, which is located in close proximity to the target volumes. It is recommended that the volume of the OAR receiving a dose of 8 Gy for the optic apparatus in SRS plans should be <0.2 cm³, and the volume receiving a dose of 10 Gy should be <0.035 cm³ [13]. Therefore, modern SRS platforms, which enable the delivery of high doses within the target volume while ensuring steep dose fall-off beyond it, offer the potential to widen the therapeutic window. In our patient, the ZAP-X gyroscopic SRS system enabled the delivery of 2500 cGy to the GTV at the 57% isodose line, while maintaining doses to OAR below recommended thresholds. Additionally, intratumoral hotspots allowed for the desired dose inhomogeneity, aligning with the core principles of SRS.

It is important to note that many patients with secreting pituitary adenomas suffer from symptoms caused by excessive hormone secretion, significantly impacting their quality of life and requiring consideration in treatment decisions [14]. In cases where patients experience severe symptoms due to elevated hormone levels, surgery may be prioritized, although various medical treatments are also viable options to consider. Pasireotide, a somatostatin analogue with multireceptor targeting, serves as an effective treatment for patients with persistent or recurring hypercortisolism post-surgery or when surgery isn't viable. However, its tolerability is debatable due to various adverse effects such as hyperglycemia and diarrhea [15]. In our patient, despite initiating pasireotide due to persistently elevated hormone levels post-surgery, it was discontinued within less than two weeks due to intolerable adverse effects.

The biological effect of SRS on hormonal hypersecretion is believed to unfold gradually, and in some cases, this process may span months or even years. Sheehan et al. [5] reported outcomes for patients with Cushing’s disease, revealing a response rate of 34% at one year, 54% at two years, 72% at three years, and 78% at five years following SRS. In our patient, a significant decrease in 24-hour urinary free cortisol level was observed within only one month after SRS. To the best of our knowledge, this represents the most rapid endocrine response after SRS reported to date. It is important to consider, however, that the rapid hormonal normalization observed in our case may have been influenced by prior medical and surgical interventions. The patient underwent two transsphenoidal surgeries and briefly received pasireotide before radiosurgery, which could have altered tumor biology or hormonal responsiveness. Nevertheless, the close temporal relationship between ZAP-X treatment and biochemical remission strongly suggests a causal association. Potential factors contributing to this rapid endocrine response may include the administration of an effective radiation dose, such as 2500 cGy, utilization of a precise SRS technique like gyroscopic radiosurgery, and the presence of hotspots within the tumor, reaching up to 4000 cGy in a single fraction. During RT, the presence of hotspots within target volumes has been shown to be associated with increased local control for various tumor types [16,17]. Therefore, while it may not be directly attributable to hotspots, it seems possible that the underlying mechanism of the rapid endocrine response observed in our patient could be related to them. However, the short follow-up duration is the main limitation of this report.

## Conclusions

To our knowledge, we report the first case of a refractory ACTH-secreting pituitary adenoma successfully treated using the vault-free ZAP-X gyroscopic SRS system. This case demonstrates that the unique design of the ZAP-X platform enables the safe delivery of a highly effective dose to the target while minimizing exposure to surrounding structures. In patients with Cushing’s disease, SRS can achieve rapid endocrine remission, although prospective studies are warranted to define the optimal dose and treatment parameters.
